# Quantifying riverbank and soil erosion risks in the upper Ghaghara river basin and their implications for flood management

**DOI:** 10.1038/s41598-025-33264-4

**Published:** 2026-01-16

**Authors:** Santosh Kumar Pandey, Saurabh Singh, Hitesh Supe, Rajnish Kaur Calay, Ram Avtar

**Affiliations:** 1 Graduate School of Environmental Science, Hokkaido University, Sapporo, 060-0810 Japan; 2 Department of Civil Engineering, Poornima University, Rajasthan, India; 3 Faculty of Environmental Earth Science, Hokkaido University, Sapporo, 060-0810 Japan; 4 Department of Building, Energy and Material Technology, The Arctic University of Norway, Narvik, Norway; 5 Department of Civil Engineering, Chennai Institute of Technology, Chennai, 600069 Tamilnadu India

**Keywords:** Riverbank erosion, Shoreline dynamics, Soil erosion risk, Flood susceptibility, DSAS, RUSLE, Environmental sciences, Hydrology, Natural hazards

## Abstract

Riverbank erosion, soil loss, and flooding are interrelated processes that critically influence river morphology, sediment dynamics, and floodplain stability. Understanding their spatial and temporal interactions is essential for effective watershed management. This study evaluates the spatio-temporal patterns of river bank change along the Upper Ghaghara River basin and investigates their relationship with regional soil erosion risk and flood susceptibility. River bank dynamics were quantified using the Digital Shoreline Analysis System (DSAS v. 6.0) applied to multi-decadal satellite imagery from 1991 to 2024, calculating key metrics including End Point Rate (EPR), Net Shoreline Movement (NSM), and Shoreline Change Envelope (SCE). Soil erosion risk was modeled using the Revised Universal Soil Loss Equation (RUSLE), incorporating rainfall erosivity, soil erodibility, slope, vegetation cover, and conservation practices, while flood susceptibility was assessed by integrating multiple hydro-geomorphic factors using the Analytic Hierarchy Process (AHP). Results indicate pronounced erosion along the right bank with an average EPR of − 20.76 m/yr and an average NSM retreat of − 687.4 m, compared to − 5.53 m/yr and − 176.7 m, respectively, on the left bank. Approximately 62.7% of right bank transects and 57.5% of left bank transects exhibit significant erosion. Soil erosion rates exceed 40 t/ha/yr in high-risk zones of the basin spatially coincident with active bank migration areas. Flood susceptibility mapping identifies 30% of the basin under high to very high flood risk, corroborated by 2022 pre- and post-flood inundation analyses showing extensive floodplain coverage. These findings highlight the synergistic impact of bank erosion, upland sediment supply, and flooding on river corridor dynamics. The integrated assessment underscores the necessity for multi-hazard management strategies to mitigate geomorphic hazards, sustain riverine ecosystems, and enhance socio-economic resilience in this vulnerable basin.

**Supplementary Information:**

The online version contains supplementary material available at 10.1038/s41598-025-33264-4.

## Introduction

Rivers are inherently dynamic ecosystems, constantly reshaped by processes such as bank erosion, flooding, and soil loss^1^. These interlinked processes operate across spatial and temporal scales and reinforce each other, influencing river course evolution, flood risk, and landscape resilience. Yet, risk assessments often address them separately, creating gaps in our understanding and management of river corridor hazards. Bank erosion occurs as flowing water undercuts the riverbank (fluvial action), and over-steepening leads to mass failures and lateral migration^2,3^. This process not only alters channel morphology but also becomes a major source of sediment and nutrients to downstream systems^4^. Meanwhile, soil erosion on upland slopes driven by rainfall, runoff, and land degradation mobilizes vast sediment volumes. These sediments are transported to river systems, altering channel hydraulics, aggrading beds, and influencing lateral stability^5^. Flooding intensifies this interconnected system. During high-discharge events, floodwaters increase hydraulic stress on banks, accelerate erosion, and redistribute sediment across floodplains. These same floodwaters can trigger landslides and gully erosion in adjacent uplands, further contributing to sediment loads and altering floodplain topography. Despite this strong interplay, assessments typically focus on one process. Bank dynamics are often mapped in isolation, while soil erosion and flood hazards are modeled separately. Only recently have integrated studies begun to emerge those combining flood susceptibility and hill-slope erosion using GIS and RUSLE^6^ but even these omit detailed channel migration analysis. Higher-resolution empirical studies underscore the importance of inclusion.

Riverbank erosion is a dominant fluvial hazard that shapes river morphology and destabilizes floodplains. Remote sensing and GIS-based approaches have been widely applied to quantify erosion hotspots and migration rates. Tha et al. (2022) mapped erosion hotspots along the Mekong River, identifying retreat rates up to 43 m yr^−^¹ and exposure of farmland and settlements^7^. Singh et al. (2023) analyzed Chambal River bank dynamics, showing that illegal sand mining accelerated retreat (~ 1.26 m yr^−^¹) compared to unmined sites ( ≈ − 0.18 to + 0.19 m yr^−^¹)^2^. Hasan et al. (2024) predicted future bankline shifts in the Meghna River using Landsat-based DSAS modeling validated with RMSE^8^. Recent innovations include machine learning: Ren et al. (2024) applied explainable AI to refine erosion risk maps in the Yangtze^9^, while Arora and Kumar (2024) demonstrated that riparian vegetation buffers can mitigate erosion in sediment-mined reaches^10^. Together, these studies underscore the importance of multi-decadal shoreline monitoring and predictive modeling in river management.

Classical experimental and field studies have deepened understanding of braided rivers. Ashmore (1991) demonstrated how slope, discharge, and sediment supply regulate braiding intensity and bed-load pulses^11^. Ashworth et al. (2000) documented braid-bar initiation and growth in the Jamuna River, showing how dune amalgamation, lateral accretion, and bar-tail elongation control bar morphology across flood cycles^12^. Nandi et al. (2022) synthesized Brahmaputra studies, highlighting process–form–vegetation interactions that dissipate energy at multiple scales^13^. Belletti et al. (2015) evaluated 53 braided reaches in the Rhône basin, showing that flood history strongly influences active channel width and island recovery^14^. A multi-decadal follow-up (Belletti et al., 2014) confirmed that high-magnitude, low-frequency (Q10) floods dominate width changes, while sediment supply plays a secondary role^15^. Using entropy-based metrics, Nandi et al. (2022) showed that immobile sand bars control planform variability, with peak flood stream power best explaining disorder levels^16^. Collectively, these studies reveal how morpho dynamics, floods, and vegetation feedbacks shape braided riverscapes.

Flooding interacts strongly with bank erosion and soil loss. Gebremichael et al. (2025) applied AHP–RUSLE in the Awash Basin (Ethiopia), finding > 50% of the basin highly flood-prone with mean soil loss of 28.6 t ha^−^¹ yr^−^¹, and strong spatial overlap between erosion and flood hotspots^17^. Chen et al. (2023) integrated RUSLE with HAND-based flood models in the Yarlung Tsangpo, identifying zones of severe soil loss coinciding with high flood risk^18^. Teng et al. (2018) projected erosion increases on the Tibetan Plateau using RUSLE coupled with climate scenarios^19^. Chen et al. (2024) combined RUSLE-TLSD and InSAR to quantify erosion linked to debris flows^20^, while Mekonnen et al. (2023) produced flood hazard maps of Ethiopia’s Awash Basin, highlighting recurrent inundation of agricultural and urban areas^21^. These studies illustrate that soil erosion and flood susceptibility are not isolated processes but co-located hazards requiring integrated assessment.

Beyond hazard quantification, management strategies have been proposed globally. Abdela et al. (2025) recommend river buffer zones, vegetation restoration, and community enforcement for Ethiopia’s Weyb River floodplain^22^. Majumdar et al. (2025) suggest adaptive riparian buffer management tailored to geomorphic and land-use contexts^23^. Rakib et al. (2024) developed a restoration framework for Bangladesh’s Old Brahmaputra, combining satellite analysis with channel geometry to design erosion-control interventions^24^. Mosselman (2025) outlines sustainable stabilization of the Brahmaputra–Jamuna using a mix of engineering and bio-engineering (spurs, bank shaping, vegetative protection). Early-warning tools^25^, such as Shampa et al. (2025), provide proactive erosion-risk identification in braided systems^26^. Together, these studies indicate that a combination of hard (revetments, spurs), soft (vegetative buffers), policy (zoning, buffer regulations), and technological (EWS, remote sensing) solutions are needed. Integrated approaches that combine structural, ecological, and community-based measures are most effective for sustainable river management.

Despite rich global research on riverbank erosion, braided river morpho dynamics, soil erosion, and floods, most studies treat these hazards separately. Integrated frameworks that couple shoreline change (DSAS), upland soil erosion (RUSLE), and flood susceptibility (AHP) remain scarce, particularly in the Upper Ghaghara Basin. This gap highlights the need for a multi-hazard approach linking lateral migration, soil erosion risk, and flood susceptibility.

Collectively, these studies provide critical insights into braided river morphology, sediment dynamics, and the co-occurrence of flood and erosion hazards across global river systems. However, most existing work has focused either on large braided systems (e.g., Brahmaputra, Jamuna, Rhône) or on single-hazard perspectives, without simultaneously linking multi-decadal riverbank migration, catchment-scale soil erosion, and flood susceptibility. This gap is particularly evident in Himalayan tributary basins such as the Upper Ghaghara, where dynamic river morphology, high monsoon variability, and intensive land use interact. Addressing this gap, the present study integrates DSAS, RUSLE, and AHP within a unified geospatial framework to provide the first multi-hazard assessment of erosion and flood risk in the Upper Ghaghara Basin.

This study aims to bridge the identified research gap by –.

Utilizing the DSAS to assess multi-decadal shoreline changes (1991–2024) along the Upper Ghaghara River (post confluence reaches of Upper Ghaghara River), focusing on metrics such as EPR, NSM, and SCE.

Applying the RUSLE to estimate mean annual soil loss across the basin, incorporating factors like rainfall erosivity, soil erodibility, slope, land cover, and conservation practices.

Conducting flood susceptibility mapping based on topographic and hydrological parameters to delineate areas at varying levels of flood risk.

This research is significant as it provides an integrated assessment of the Upper Ghaghara Basin geomorphic and hydrological dynamics, offering a comprehensive understanding of the interactions between shoreline change, soil erosion, and flooding. By identifying areas of high composite risk, the study informs targeted interventions for riverbank stabilization, soil conservation, and flood mitigation. The findings contribute to the development of sustainable watershed management strategies that enhance resilience to natural hazards and support the socio-economic well-being of communities dependent on the river system.

The novelty of this study lies in its integrated application of established geospatial techniques—DSAS for shoreline dynamics, RUSLE for soil erosion risk, and AHP for flood susceptibility—within a single unified framework. By applying these widely recognized methods together on multi-decadal datasets, the study provides a holistic understanding of the interconnected processes of bankline migration, upland soil erosion, and flood susceptibility in the Upper Ghaghara Basin. The identification of composite risk hotspots adds value by highlighting zones where multiple hazards overlap, offering a reproducible workflow that can also inform similar assessments in other riverine landscapes.

## Materials and methods

### Study area and rationale

**Fig. 1:**
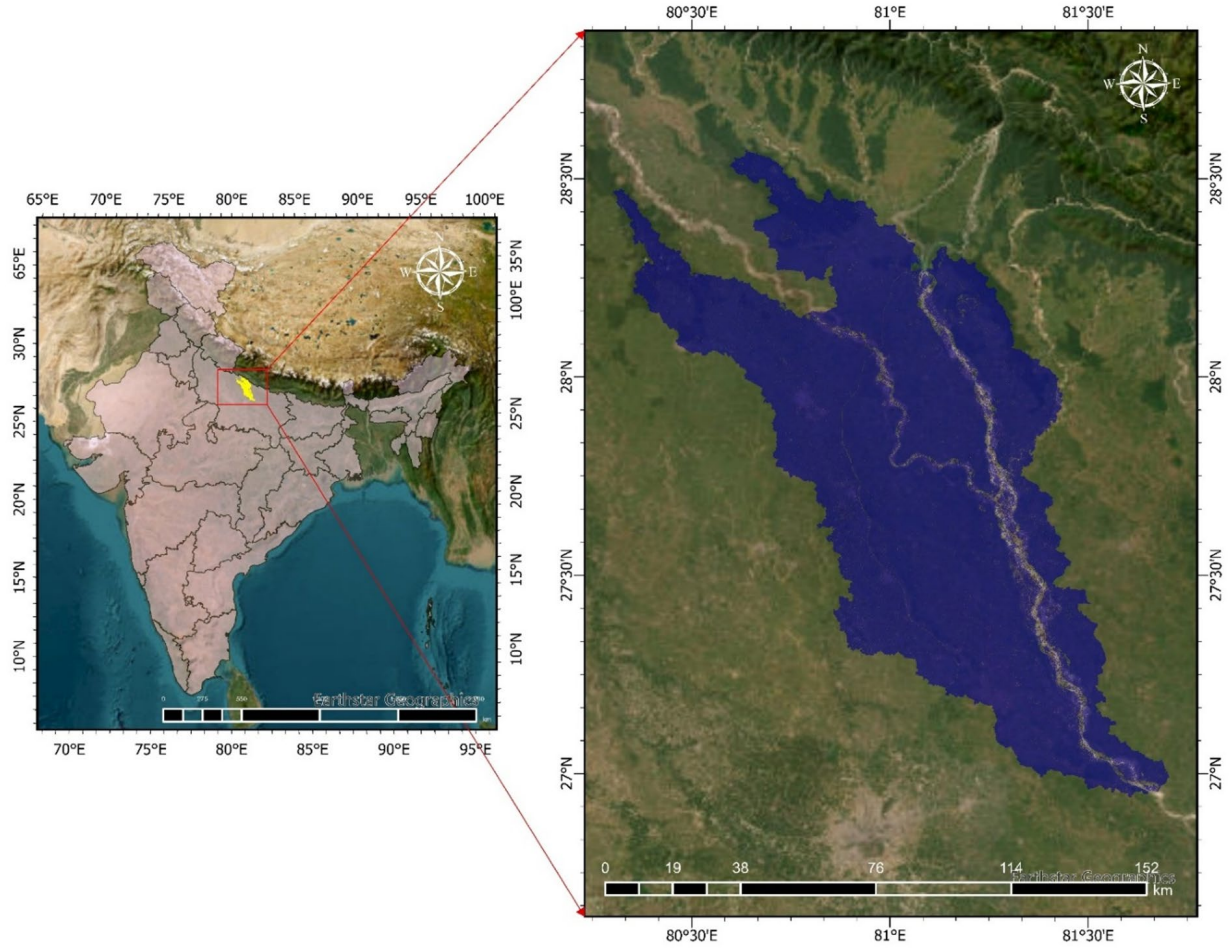
Map of the Upper Ghaghara River Basin generated using ArcGIS Pro 3.2 (licensed version; https://www.esri.com/en-us/arcgis/products/arcgis-pro). The map shows major tributaries, elevation gradients, and administrative boundaries. India country boundary shapefile was obtained from the Survey of India online maps portal (https://onlinemaps.surveyofindia.gov.in/).

The study focuses on the Upper Ghaghara Basin, situated between approximately 27.5°N and 28.5°N latitude and 81.5°E and 82.5°E longitude, covering a total area of approximately 9262.70 km² (Fig. 1). This segment of the Ghaghara River, also known as the Karnali River in Nepal, is a significant left-bank tributary of the Ganges. Originating from the Tibetan Plateau, it traverses Nepal and enters India, flowing through the states of Uttar Pradesh and Bihar before merging with the Ganges below Chapra.

The Upper Ghaghara Basin is characterized by its dynamic geomorphic activity, including significant lateral erosion and sediment transport. These processes are influenced by the river’s high sinuosity and the presence of unconsolidated sediments such as sand, silt, and clay^27^. Studies have documented substantial lateral erosion, with river migration observed in various locations along the basin^28^. The river’s morphology and sediment dynamics are further shaped by the seasonal monsoon rains and glacial melt from the Himalayas, contributing to its high sediment load and variability in discharge^29^.

The availability of multi-temporal satellite imagery, such as Landsat and Sentinel data, facilitates the application of the DSAS to assess river bank line dynamics over time^30^. Moreover, the basin’s significance is underscored by its role in regional hydrology and its susceptibility to flooding and erosion, which impact local communities and agriculture^31^.

### Data acquisition and preprocessing

To enable an integrated analysis, multi-source datasets were compiled and processed within a unified spatial framework. High-resolution satellite imagery (Landsat, Sentinel-2, IRS) and topographic maps were georeferenced to WGS 84 / UTM Zone 44 N. Shoreline positions for nine time points (1991–2024) were digitized at 1:10,000 scale, while RUSLE input layers (rainfall, soil, slope, land cover, management practice) were prepared at 30 m spatial resolution (Table 1).

**Table 1:** Summary of datasets, their sources, Spatial resolutions, and specific uses in the analysis for shoreline dynamics and soil erosion modeling in the upper Ghaghara river Basin.

Data type	Spatial resolution	Source & link	Use in analysis
Landsat imagery	30 m	USGS Landsat Collection 2 https://landsat.gsfc.nasa.gov/data/data-access/ (landsat.gsfc.nasa.gov)	Shoreline digitization; baseline land-cover mapping
Sentinel-2 MSI imagery	10–20 m	Copernicus Open Access Hub https://scihub.copernicus.eu/ (eodatahub.com, Sentinel Online)	High-resolution shoreline delineation; LULC analysis
IRS imagery	~ 5–20 m	Bhuvan / NRSC & ISRO Bhoonidhi portal https://bhoonidhi.nrsc.gov.in/ (bhoonidhi.nrsc.gov.in, National Road Safety Council)	Detailed shoreline feature mapping
Topographic maps	1:50,000 scale	Survey of India / Map Portal India https://onlinemaps.surveyofindia.gov.in/	Baseline georeferencing for shoreline digitization
IMD rainfall data	0.25° grid (~ 25 km)	IMD Pune gridded data https://imdpune.gov.in/cmpg/Griddata/Rainfall_25_NetCDF.html (imdpune.gov.in, imdpune.gov.in)	Rainfall erosivity (R factor for RUSLE)
NBSS & LUP soil maps	1:250,000–1:50,000 scale	NBSS & LUP Geoportal (Bhoomi) https://bhoomigeoportal-nbsslup.in/ (bhoomigeoportal-nbsslup.in, isric.org)	Soil erodibility (K factor for RUSLE)
SRTM DEM	30 m	USGS/NASA Shuttle Radar Topography Mission https://earthexplorer.usgs.gov/ (NASA Earthdata, USGS)	Slope length–steepness (LS factor for RUSLE)
Field survey & Sentinel-2 LULC	30 m raster	Field observations; Sentinel-2 data as above	Cover-management (C) and practice (P) factors (RUSLE)

### Quantifying shoreline dynamics

#### Transect generation and DSAS metrics

Bankline positions were analyzed using the DSAS version 6.0 a GIS-based tool designed for quantifying riverbank migration and morphological changes^32^. A fixed baseline was established along the historic river corridor, from which 1,767 orthogonal transects spaced at 100–500 m intervals were generated to intersect bankline positions digitized at nine time points (1991–2024). Key DSAS metrics calculated include EPR, NSM, SCE, and LRR, defined as:

$$\:EPR=\frac{{\mathrm{D}}_{\mathrm{e}\mathrm{n}\mathrm{d}\:}-{\mathrm{D}}_{\mathrm{s}\mathrm{t}\mathrm{a}\mathrm{r}\mathrm{t}\:}\text{}\text{}}{{\mathrm{T}}_{\mathrm{e}\mathrm{n}\mathrm{d}\:}-{\mathrm{T}}_{\mathrm{s}\mathrm{t}\mathrm{a}\mathrm{r}\mathrm{t}\:}}$$

This yields the average annual rate of bank movement (m/yr) over the study period^33^.

$$\:\mathrm{N}\mathrm{S}\mathrm{M}={\mathrm{D}}_{\mathrm{e}\mathrm{n}\mathrm{d}}-{\mathrm{D}}_{\mathrm{s}\mathrm{t}\mathrm{a}\mathrm{r}\mathrm{t}}$$

Total positional shift (in meters) between the first and last shoreline^34^.

$$\:\mathrm{S}\mathrm{C}\mathrm{E}={\mathrm{D}}_{\mathrm{m}\mathrm{a}\mathrm{x}}-{\mathrm{D}}_{\mathrm{m}\mathrm{i}\mathrm{n}}$$

Represents the full range of bank oscillation across the study period.

$$\:\mathrm{L}\mathrm{R}\mathrm{R}=\frac{\sum\:({\mathrm{t}}_{\mathrm{i}}-\overline{\mathrm{t}}\:)({\mathrm{d}}_{\mathrm{i}}-\overline{\mathrm{d}}\:)}{\sum\:{({\mathrm{t}}_{\mathrm{i}}-\overline{\mathrm{t}}\:)}^{2}}$$

Provides a more robust trend across multiple observations.

### Flood susceptibility mapping using AHP

Flood susceptibility in the Upper Ghaghara River Basin was assessed by integrating multiple hydro-geomorphic factors using the AHP within a GIS framework implemented in Google Earth Engine (GEE). Five flood-conditioning factors were selected based on their hydrological and geomorphic significance and data availability: elevation (USGS SRTM DEM, 30 m), slope (derived from DEM), annual precipitation (CHIRPS, 2020), distance to major rivers (Hydro SHEDS Free-Flowing Rivers), and land use/land cover (MODIS MCD12Q1, 2020).

#### Factor standardization

Each factor was reclassified into an ordinal flood risk scale from 1 (very low risk) to 5 (very high risk) using threshold-based expressions:

$${S_i}=\left\{ {\begin{array}{*{20}{l}} {5,}&{{f_i}\left( {x,y} \right) \in \;{\mathrm{highest}}\;{\mathrm{risk}}\;{\mathrm{class}}} \\ {4,}&{{f_i}\left( {x,y} \right) \in \;{\mathrm{high}}\;{\mathrm{risk}}\;{\mathrm{class}}} \\ {3,}&{{f_i}\left( {x,y} \right) \in \;{\mathrm{moderate}}\;{\mathrm{risk}}\;{\mathrm{class}}} \\ {2,}&{{f_i}\left( {x,y} \right) \in \;{\mathrm{low}}\;{\mathrm{risk}}\;{\mathrm{class}}} \\ {1,}&{{f_i}\left( {x,y} \right) \in \;{\mathrm{very}}\;{\mathrm{low}}\;{\mathrm{risk}}\;{\mathrm{class}}} \end{array}} \right.$$

  

where Si (x, y) is the standardized score for factor i at pixel (x, y), and fi (x, y) is the original factor value.

#### Weight derivation via AHP

Pairwise comparisons of factor importance were performed based on expert knowledge and literature [e.g., 5,6], forming a comparison matrix A:

$$\:A={[a}_{ij}]\:\:\:\:\:{a}_{ij}=importance\:of\:factor\:i\:over\:j$$

The normalized principal eigenvector of A yielded factor weights wi​, satisfying $\:{\sum\:}_{i=1}^{n}{w}_{i}=1$. Consistency Ratio (CR) was computed to ensure logical consistency:

$${\mathrm{CR}}=\frac{{{\lambda _{\hbox{max} }} - n}}{{\left( {n - 1} \right) \cdot {\mathrm{RI}}}}<0.10$$

Where λmax​ is the maximum eigenvalue, n is the number of factors, and RI is the random index. A CR value of 0.06 was obtained, which is below the widely accepted threshold of 0.10^35^, confirming logical consistency of the comparisons. To assess robustness, each factor weight was perturbed by ± 10% while maintaining normalization. The resulting Flood Susceptibility Index (FSI) maps were compared with the baseline. Results showed that the spatial extent of the High and Very High flood susceptibility zones changed by < 5%, and pixel-wise agreement remained above 90% (κ = 0.82). This indicates that the AHP-based flood susceptibility mapping is robust to small variations in weight assignment, in line with recent flood risk studies. The final weights applied were according to Table 2.

**Table 2:** Final AHP factor weights.

Factor	Weight (wi)
Elevation	0.08
Slope	0.11
Annual Precipitation	0.02
Distance to River	0.03
Land Use/Land Cover	0.21

#### Flood susceptibility index (FSI) computation

The Flood Susceptibility Index was computed via weighted linear combination of standardized factors:

$$FSI\left( {x,y} \right)=\sum\limits_{{i=1}}^{n} {{w_i} \times {S_i}\left( {x,y} \right)}$$

  

where *n* = 5 is the number of factors.

The continuous FSI raster was further classified into five discrete susceptibility zones:

$$Zone\left( {x,y} \right)=\left\{ {\begin{array}{*{20}{l}} {1,}&{{\mathrm{FSI<1}}{\text{.5 }}\;\left( {{\mathrm{Very}}\;{\mathrm{Low}}} \right)} \\ {2,}&{1.5 \leqslant {\mathrm{FSI}}<2.5\;\left( {{\mathrm{Low}}} \right)} \\ {3,}&{2.5 \leqslant {\mathrm{FSI}}<3.5\;\left( {{\mathrm{moderate}}} \right)} \\ {4,}&{3.5 \leqslant {\mathrm{FSI}}<4.5} \\ {5,}&{{\mathrm{FSI}} \geqslant {\mathrm{4}}{\mathrm{.5}}\;\left( {{\mathrm{Very}}\;{\mathrm{high}}} \right)} \end{array}} \right.$$

  

All spatial processing and overlay analyses were conducted using GEE, clipping outputs to the study boundary for precise spatial delineation.

### Modeling soil erosion risk

#### RUSLE implementation

Soil erosion was modeled using the RUSLE, which estimates mean annual soil loss (A, t/ha/yr) per grid cell as^36^:

$$\:\mathrm{A}=\mathrm{R}\times\:\mathrm{K}\times\:\mathrm{L}\mathrm{S}\times\:\mathrm{C}\times\:\mathrm{P}$$

Where:

R = Rainfall erosivity (derived from mean annual precipitation, IMD data, using the empirical relation *R* = 0.5×P. This simplified approach is widely applied in data-scarce regions but carries limitations, as it may under- or overestimate erosivity relative to intensity-based calculations. For example, Cruz et al. (2025) demonstrated that the *R* = 0.5 × P method can significantly diverge from intensity-based estimates in Southeast Asia, sometimes underestimating erosivity by 15–25%^37^. To account for this, we conducted a sensitivity check, noting that erosivity estimates may vary by ± 20–30% when compared with intensity-derived methods.

K = Soil erodibility (from NBSS&LUP soil maps, incorporating texture and organic matter)^38^.

LS = Slope length-steepness (calculated from SRTM DEM using the standard RUSLE formula)^39^.

C = Cover-management factor (assigned by land cover class from Sentinel-2).

P = Support practice (based on field mapping; values range from 1.0 for no practice to 0.5 for contour farming)^40^.

All factor layers were resampled to a common 30 m grid and combined using raster calculation. Resulting soil loss estimates were classified into five risk zones (Slight < 10, Moderate 10–20, High 20–30, Very High 30–40, Severe > 40 t/ha/yr).

### Establishing the shoreline–erosion relationship

#### Spatial overlay and hotspot analysis

To explicitly link shoreline mobility and soil erosion, all DSAS-derived metrics (EPR, NSM, SCE) were spatially overlaid with the RUSLE soil loss map using GIS. Zones with both high shoreline change (e.g., top quartile of SCE or EPR) and severe soil loss (> 40 t/ha/yr) were mapped as composite risk “hotspots.”

#### Integrated workflow

This integrated approach (shown in Table 3) allows for direct comparison and correlation between riverbank dynamics and catchment-level soil erosion, capturing feedback such as:

Areas where active channel migration directly amplifies landscape soil loss by bank collapse and sediment remobilization.

Sites where severe upland erosion contributes to fluvial sediment load, potentially altering channel behavior downstream.

**Table 3:** Key analytical Steps, Parameters, and data Sources.

Step	Metric / Formula	Input Data / Source
Shoreline change	EPR, NSM, SCE, LRR	DSAS transect analysis of digitized shorelines (1991–2024), ArcGIS Pro 3.2
Rainfall erosivity	*R* = 0.5 × PR	IMD 0.25° daily gridded rainfall data, NetCDF format, 1901–2022: https://imdpune.gov.in/cmpg/Griddata/Rainfall_25_NetCDF.html (imdpune.gov.in)
Soil erodibility	Lab/field-based estimation	NBSS & LUP soil survey via Bhoomi Geoportal: https://bhoomigeoportal-nbsslup.in/ (bhoomigeoportal-nbsslup.in)
Slope length-steepness	LS via DEM-based formula	SRTM 1″ (~ 30 m) DEM via USGS EarthExplorer: https://earthexplorer.usgs.gov/ (USGS, USGS)
Cover-management (C)	Land cover-based assignment	Sentinel-2 MSI imagery via Copernicus Open Access Hub: https://scihub.copernicus.eu/
Support practice (P)	Field-assigned values	Field survey; Survey of India topographic maps via https://onlinemaps.surveyofindia.gov.in/
Soil loss estimation	A = R × K × LS × C × P	RUSLE raster overlay using combined C, K, LS, P, and R factors
Shoreline–erosion overlay	Spatial intersection & hotspot mapping	GIS spatial analysis using ArcGIS Pro 3.2 and DSAS

### Data analysis and visualization

All geospatial analyses, including data processing, spatial overlays, and map generation, were conducted using ArcGIS Pro 3.2 and QGIS 3.28. The analysis produced detailed transect-based trend graphs that illustrate temporal variations in key shoreline metrics such as EPR, NSM, SCE each plotted against their respective transect identifiers. In addition, composite risk maps were generated to integrate zones exhibiting significant shoreline change with areas classified as having severe soil erosion, thereby identifying spatial hotspots of geomorphic vulnerability. Statistical summaries were also compiled to quantify the proportions of erosional versus accretional transects, average rates of shoreline movement, and the spatial distribution of erosion risk classes across the study area. These visualization outputs provide a comprehensive framework for interpreting the spatial and temporal dynamics of riverbank processes and their associated hazards.

Although the integrated DSAS–RUSLE–AHP framework provides a reproducible and data-driven workflow, several limitations must be acknowledged. First, the rainfall erosivity factor (R) in RUSLE was derived using the simplified *R* = 0.5 × P relation. While widely applied in data-scarce regions, this method may under- or overestimate erosivity relative to intensity-based formulations. Sensitivity analysis indicated that soil loss estimates may vary by ± 20–30% compared to intensity-derived values, consistent with earlier findings in tropical and monsoon basins (e.g., Cruz et al., 2025). Second, the AHP relies on expert judgment for weight assignment. Although the Consistency Ratio (CR = 0.06) confirmed logical consistency and perturbation tests (± 10% weight variation) altered high-risk zones by less than 5%, some subjectivity remains. Third, while the DSAS analysis benefits from multi-decadal satellite records, uncertainties in shoreline digitization (e.g., mixed pixels, seasonal water levels) may affect absolute erosion rates. Despite these constraints, the triangulation of multiple methods, sensitivity testing, and comparison with observed 2022 flood extents provide confidence in the robustness of our results.

## Results

### Comparative shoreline change dynamics of the Ghaghara riverbanks

The DSAS analysis of shoreline changes along the ‘post confluence reaches of Upper Ghaghara River’, referred as ‘Upper Ghaghara River’ in this study; reveals marked contrasts in the magnitude and direction of bank movement between the right and left banks. On the right bank, which was analyzed using 840 transects spaced at 100 m intervals, the SCE averaged 3,545.8 m, ranging from a minimum of 101.6 m (Transect 641) to a maximum of 7,347.8 m (Transect 276). Net Shoreline Movement (NSM) on this bank averaged − 687.4 m, with 62.7% of transects exhibiting net erosion (maximum retreat of − 4,624.8 m at Transect 528) and the remaining 37.3% showing accretion (maximum advance of 3,722.6 m at Transect 276). The EPR averaged − 20.76 m/yr (± 0.44 m/yr uncertainty), with erosional transects retreating at an average of − 54.60 m/yr and accretional transects advancing at 36.22 m/yr; maximum rates were − 140.14 m/yr (Transect 528) and 112.80 m/yr (Transect 276), respectively, and over 60% of transects showed statistically significant erosion. Both the Linear Regression Rate (LRR) and the Weighted Linear Regression (WLR) yielded an average trend of − 15.86 m/yr, with 63.1% of transects retreating overall.

In contrast, the left bank analysis based on 927 equally spaced transects returned a lower average SCE of 3,246.5 m (min. 21.8 m at Transect 933; max. 7,607.7 m at Transect 282). Its NSM averaged − 176.7 m, with 57.5% of transects receding (maximum − 3,596.6 m at Transect 484) and 42.5% advancing (maximum 3,656.7 m at Transect 539). The EPR on the left bank averaged − 5.53 m/yr (± 0.03 m/yr), with average erosional and accretional rates of − 43.79 m/yr and 46.23 m/yr extremes of − 108.99 m/yr (Transect 484) and 110.81 m/yr (Transect 539)—and statistically significant changes in roughly 57% (erosion) and 42% (accretion) of transects. The LRR showed a small positive mean (0.92 m/yr) while the WLR yielded − 1.78 m/yr, reflecting near-balance between erosion and accretion when all dates are considered, with just over half of transects retreating under regression analysis.

These results underscore that both banks are predominantly erosional, but the right bank experiences more intense and extensive retreat. Table 4 summarizes these key metrics for direct comparison.

**Table 4:** Summary of shoreline change metrics for the right and left banks of the upper Ghaghara River.

Metric	Right Bank	Left Bank
Number of Transects	840	927
Avg SCE (m)	3,545.8	3,246.5
Max SCE (m)	7,347.8 (ID 276)	7,607.7 (ID 282)
Min SCE (m)	101.6 (ID 641)	21.8 (ID 933)
Avg NSM (m)	–687.4	–176.7
% Erosional Transects (NSM)	62.7%	57.5%
Avg EPR (m/yr)	–20.76 ± 0.44	–5.53 ± 0.03
Avg Erosional EPR (m/yr)	–54.60	–43.79
Avg Accretional EPR (m/yr)	36.22	46.23
Max Erosional EPR (m/yr)	–140.14 (ID 528)	–108.99 (ID 484)
Max Accretional EPR (m/yr)	112.80 (ID 276)	110.81 (ID 539)
% Significant Erosion (EPR)	61.7%	56.9%
LRR Mean (m/yr)	–15.86	0.92
WLR Mean (m/yr)	–15.86	–1.78
% Erosional Transects (LRR)	63.1%	52.7%

While the physical processes of bankline migration are quantified via SCE, NSM, and EPR metrics, these dynamics carry important downstream consequences. In similar river systems, riverbank migration has been shown to damage agricultural lands, erode homesteads, and impair infrastructure such as roads and embankments^41–43^. Outlier transects with values much higher than basin averages likely represent reaches where rapid erosion or deposition is most active. These reaches are probably responsible for disproportionate land loss or infrastructure risk, making them key targets for monitoring and mitigation.

Outlier transects with extreme retreat (here, EPR ≤ − 100 m yr^−^¹) or very large lateral change (SCE ≥ 5,000 m) are designated high-priority hotspots. Composite hotspots where rapid erosion coincides with severe upslope soil loss are included in this class. These sites require immediate on-site actions: (i) Riparian vegetation and bioengineering (e.g., brush layering, live staking, coir/log elements) to stabilize outside bends and toes; (ii) Localized early warning and inspections; (iii) Temporary sand-mining controls where hotspots intersect extraction; (iv) Prioritization near critical assets (e.g., segments within ~ 200 m of settlements, bridges, or intakes).Highlighting these actionable hotspots gives stakeholders a practical pathway to sequence resources and reduce vulnerability in the most dynamic river segments.

These findings suggest that mapping high-change reaches (where SCE/NSM/EPR are elevated) is not only of academic interest but can be directly actionable: they help prioritize areas at risk for agricultural loss, settlement encroachment, and infrastructure vulnerability.

### Spatial and transect-wise variation of shoreline dynamics along the left bank

**Fig. 2:**
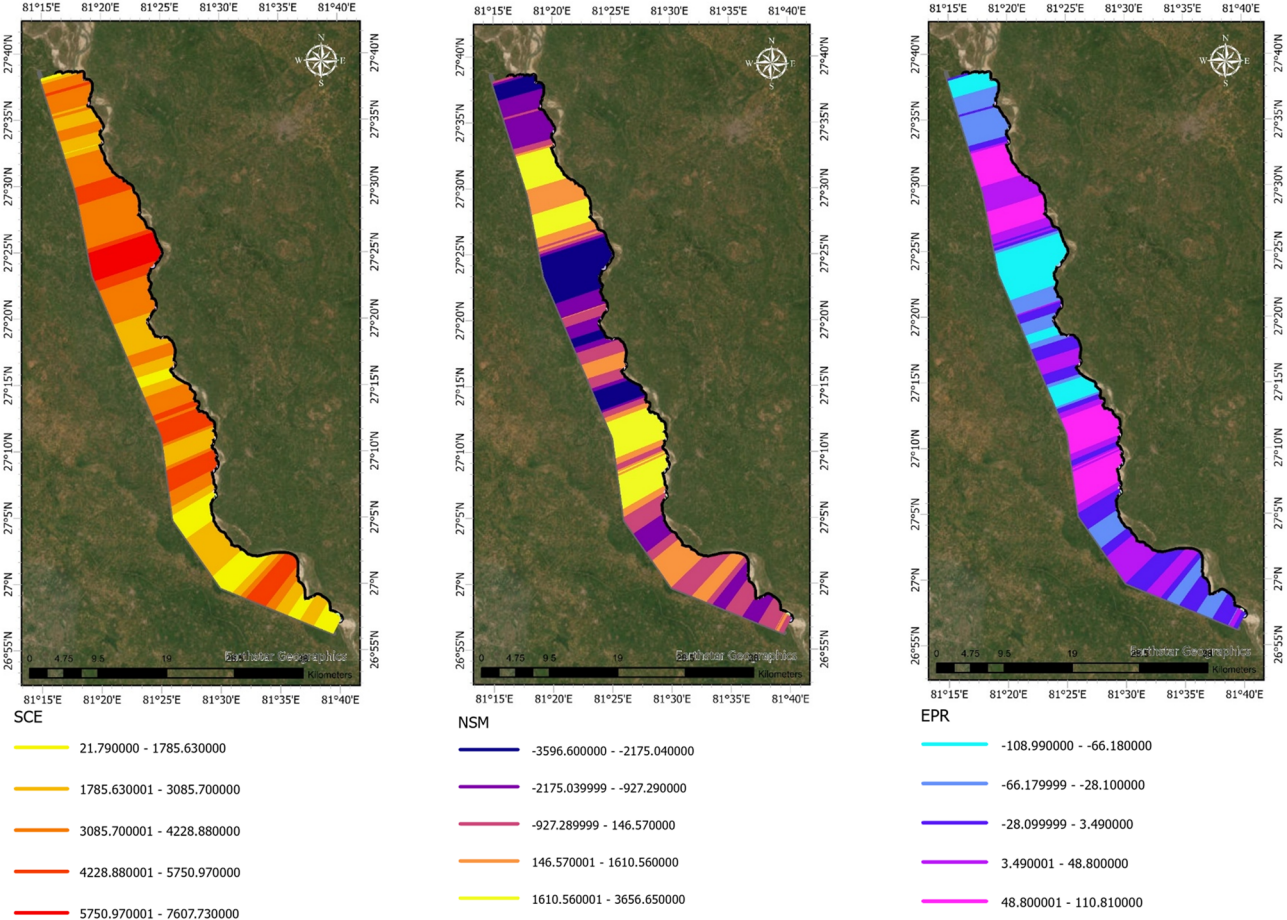
Spatio-temporal variation in shoreline dynamics along the left bank of the Upper Ghaghara River (1991–2024). Metrics (SCE, NSM, EPR) were computed using DSAS (v6.0; https://code.usgs.gov/cch/dsas) and resulting maps were exported to ArcGIS Pro 3.2 (licensed version; https://www.esri.com/en-us/arcgis/products/arcgis-pro). Warmer colors in SCE indicate higher migration, purple in NSM shows erosion while yellow denotes accretion, and blue in EPR reflects high erosion rates while pink–violet indicate stability or accretion.

The spatio-temporal analysis of shoreline dynamics along the left bank of the Upper Ghaghara River reveals pronounced variability in channel migration between 1991 and 2024. Figure 2 shows the spatial distribution of key shoreline change metrics. The SCE ranges widely along the river reach, with the highest lateral oscillations concentrated in the central sector between approximately 27°30′ N and 27°55′ N, where SCE values exceed 5,000 m. Similarly, the Net Shoreline Movement (NSM) map highlights extended zones of significant bank retreat, with maximum negative displacements reaching over − 3,500 m. The EPR map further quantifies these changes temporally, showing annual erosion rates of up to − 100 m/yr concentrated in the same central reach. Upstream and downstream regions display comparatively subdued dynamics, with lower SCE values (ranging between 1,800 and 4,200 m), mixed NSM patterns, and EPR rates generally confined within ± 10 m/yr.

**Fig. 3:**
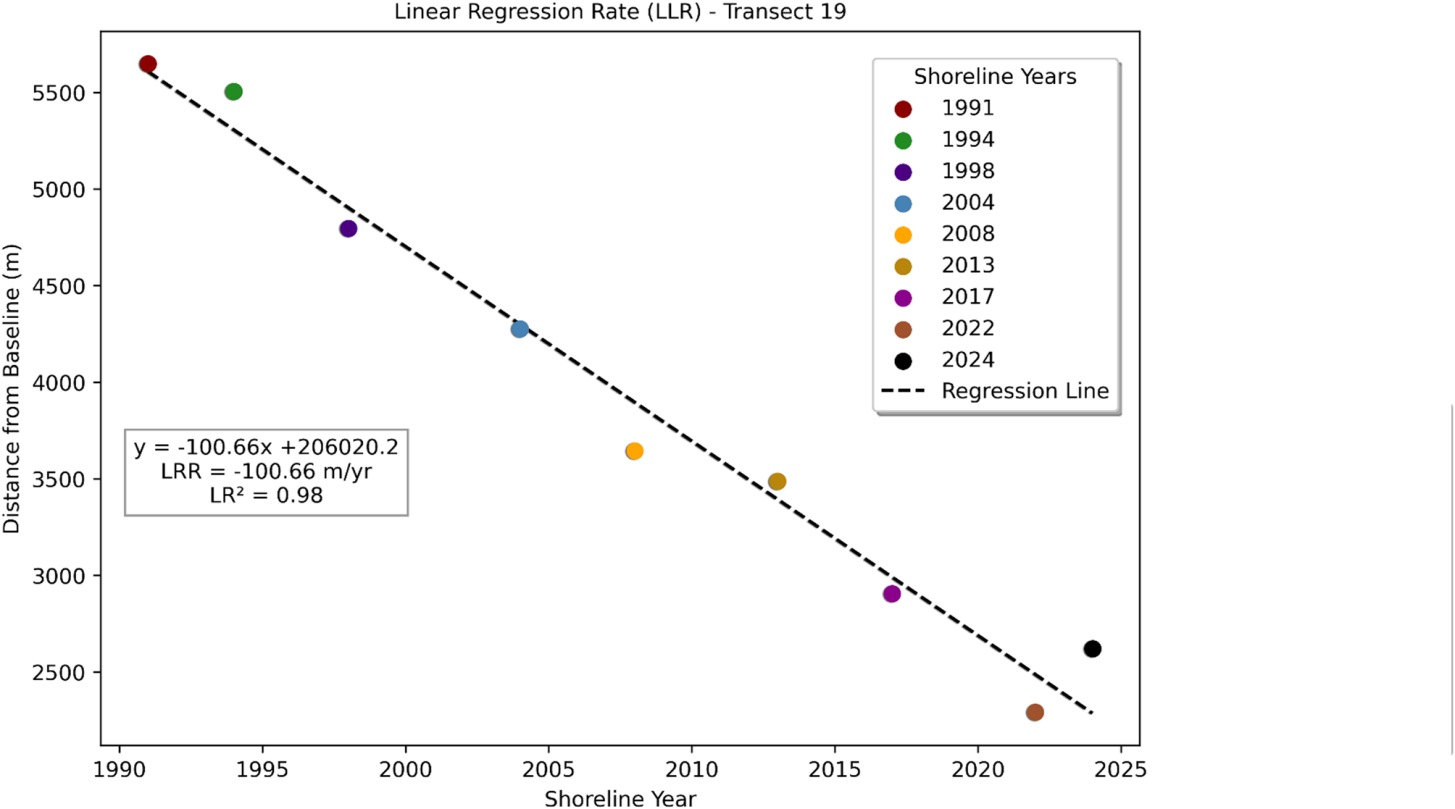
Left-bank shoreline distance from the baseline (1991–2024) with a linear erosion trend of − 100.66 m yr^−^¹ (R² = 0.98). Plot created in Python using Matplotlib (https://matplotlib.org).

Figure 3 shows the distance of the left-bank shoreline from the baseline between 1991 and 2024 for a representative transect of the Upper Ghaghara River. The dashed line represents the linear regression trend, revealing a strong and consistent pattern of shoreline retreat, with an average erosion rate of − 100.66 m/yr (LRR) and a very high correlation (R² = 0.98), indicating rapid and persistent bank erosion at this location.

The regression equation, quantifies this trend over the 33-year period. This high rate of erosion highlights significant geomorphic instability and underscores the need for targeted riverbank management in this vulnerable zone.

Such persistent erosion can lead to substantial loss of agricultural land, threaten local infrastructure, and alter the natural course of the river. The consistency in retreat rates suggests that erosional forces such as strong river currents and seasonal floods have dominated this stretch throughout the study period. Proactive intervention and restoration efforts are therefore critical to reduce future risks and sustain the socio-economic value of the floodplain.

**Fig. 4:**
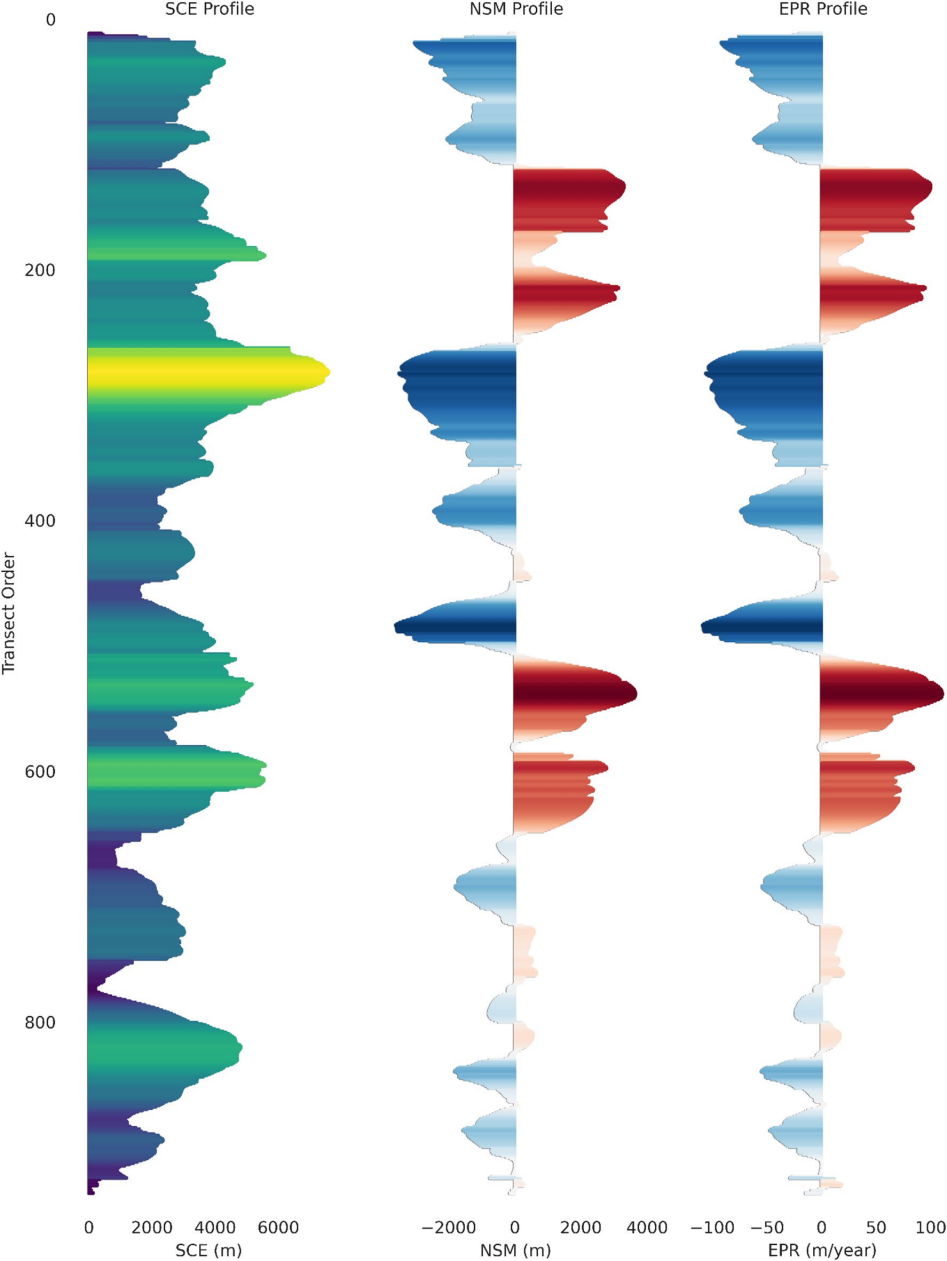
Transect-wise variation in left-bank shoreline dynamics along the Upper Ghaghara River (1991–2024). Metrics (SCE, NSM, EPR) were computed using DSAS (v6.0; https://code.usgs.gov/cch/dsas). Color gradients represent erosion (red tones, negative NSM/EPR) and accretion (blue tones, positive NSM/EPR), with higher magnitudes indicating zones of greater geomorphic instability.

Complementing these spatial observations, Fig. 4 provides a transect-wise quantification of shoreline dynamics. The SCE profile demonstrates pronounced peaks at transect IDs roughly between 250 and 350 and 500–650, aligning with the central high-mobility zone identified in Fig. 2. The NSM graph indicates that approximately two-thirds of the transects experienced net erosion, with negative displacements of − 2,000 to − 3,500 m prevalent in the mid-reach transects. Correspondingly, the EPR profile highlights the highest erosion rates (–100 to − 50 m/yr) concentrated in these transects, while positive rates of + 80 to + 100 m/yr correspond to localized accretional zones near the river’s upstream and downstream limits.

Taken together, these spatial and temporal analyses clearly demonstrate that the central segment of the left bank is the most geomorphically active, exhibiting the highest rates and magnitudes of channel migration and erosion. These findings provide critical evidence for prioritizing riverbank stabilization and targeted management interventions in this vulnerable corridor.

### Spatial and transect-wise variation of shoreline dynamics along the right bank

**Fig. 5:**
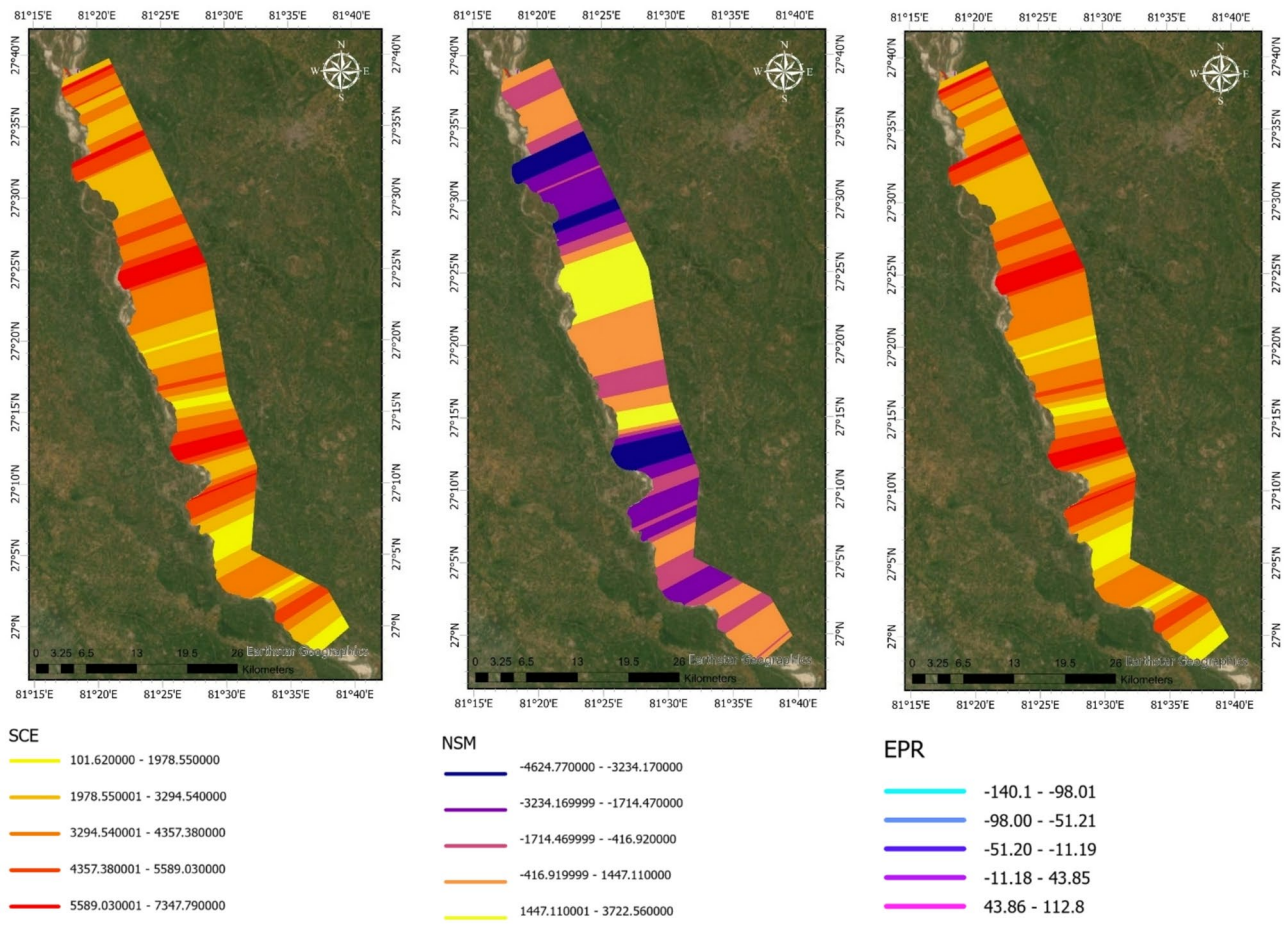
Spatio-temporal variation in shoreline dynamics along the right bank of the Upper Ghaghara River (1991–2024). Metrics (SCE, NSM, EPR) were computed using DSAS (v6.0; https://code.usgs.gov/cch/dsas) and resulting maps were exported to ArcGIS Pro 3.2 (licensed version; https://www.esri.com/en-us/arcgis/products/arcgis-pro). Warmer colors in SCE indicate higher migration, purple in NSM shows erosion while yellow denotes accretion, and blue in EPR reflects high erosion rates while pink–violet indicate stability or accretion.

**Fig. 6:**
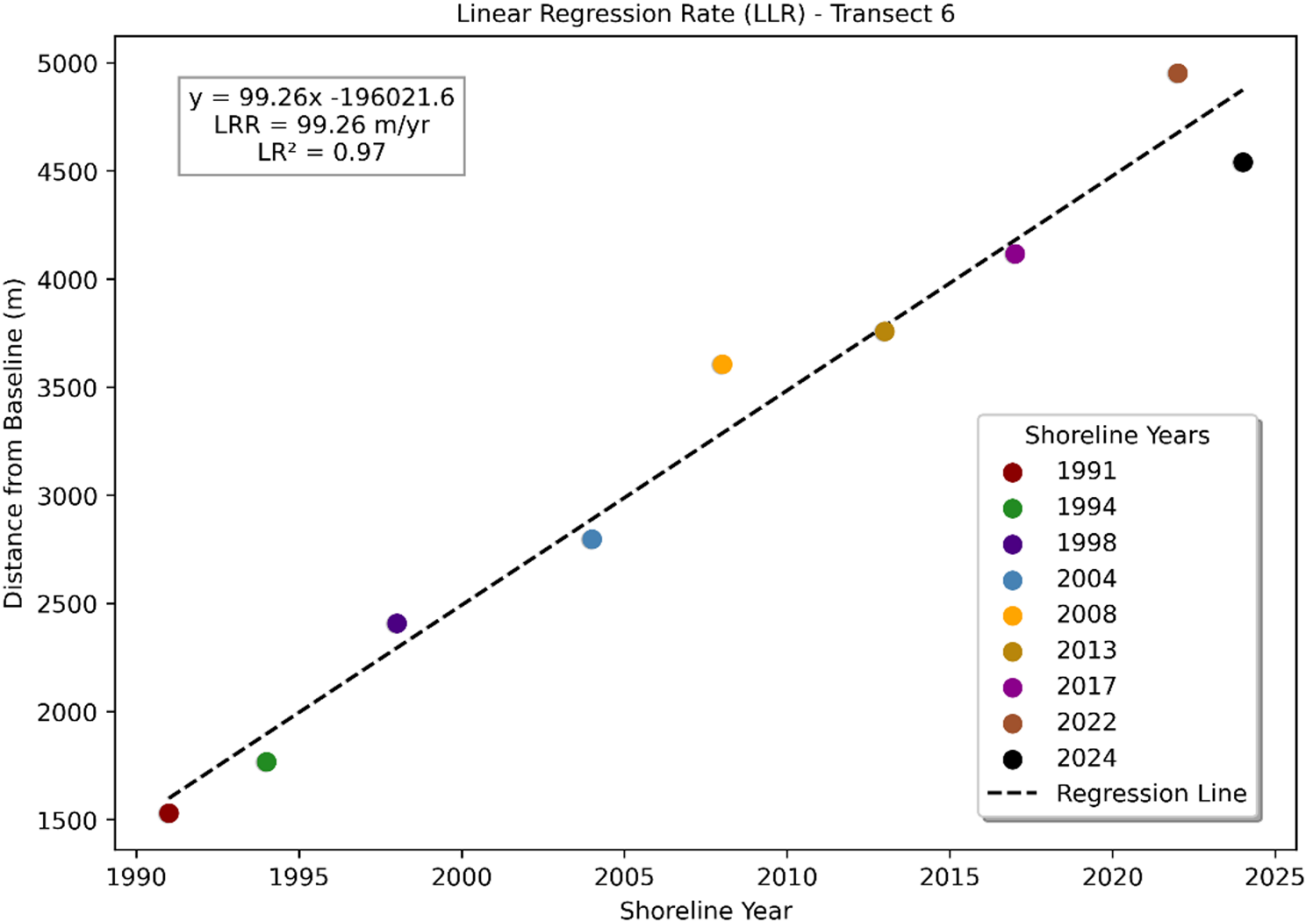
Right-bank shoreline distance from the baseline (1991–2024), showing a linear accretion trend of + 99.26 m yr^−^¹ (R² = 0.97). The plot was generated using Matplotlib in Python (https://matplotlib.org).

The spatial distribution of shoreline change along the right bank of the Upper Ghaghara Riverfrom 1991 to 2024 is shown in Fig. 5, with corresponding transect-wise variation presented in Fig. 6. SCE map shows extensive lateral oscillations across the entire study reach, with values ranging from approximately 100 m to over 7,300 m. High SCE values are notably concentrated in the mid-section of the river between approximately 27°15′ N and 27°45′ N, where the shoreline exhibits frequent and large-scale movement. Net Shoreline Movement (NSM) on the right bank reveals spatially distinct erosional and depositional zones. Large contiguous stretches of net retreat are observed, with maximum displacements exceeding − 4,600 m in the mid-section. Conversely, areas of net accretion are interspersed, particularly near the downstream end, with maximum advances approaching 3,700 m. The EPR map quantifies these spatial patterns temporally, showing that annual erosion rates of up to − 140 m/yr occur predominantly in the central segments of the right bank. Accretional rates reach as high as + 113 m/yr, with depositional zones scattered primarily toward the river’s downstream reaches.

Figure 6 shows the distance of the right-bank shoreline from the baseline between 1991 and 2024 for a representative transect of the Upper Ghaghara River. The dashed line represents the linear regression trend, indicating a consistent pattern of shoreline advance. The regression equation, $\:y=99.26x-196021.6$, reveals an average accretion rate (LRR) of + 99.26 m/yr, with a strong correlation (R^2^=0.97). This result demonstrates substantial and persistent right-bank accretion at this location over the 33-year period, in contrast to the retreat observed on the opposite bank. The magnitude and consistency of the advance suggest localized sediment deposition and favorable hydrodynamic conditions promoting bank growth in this reach.

**Fig. 7:**
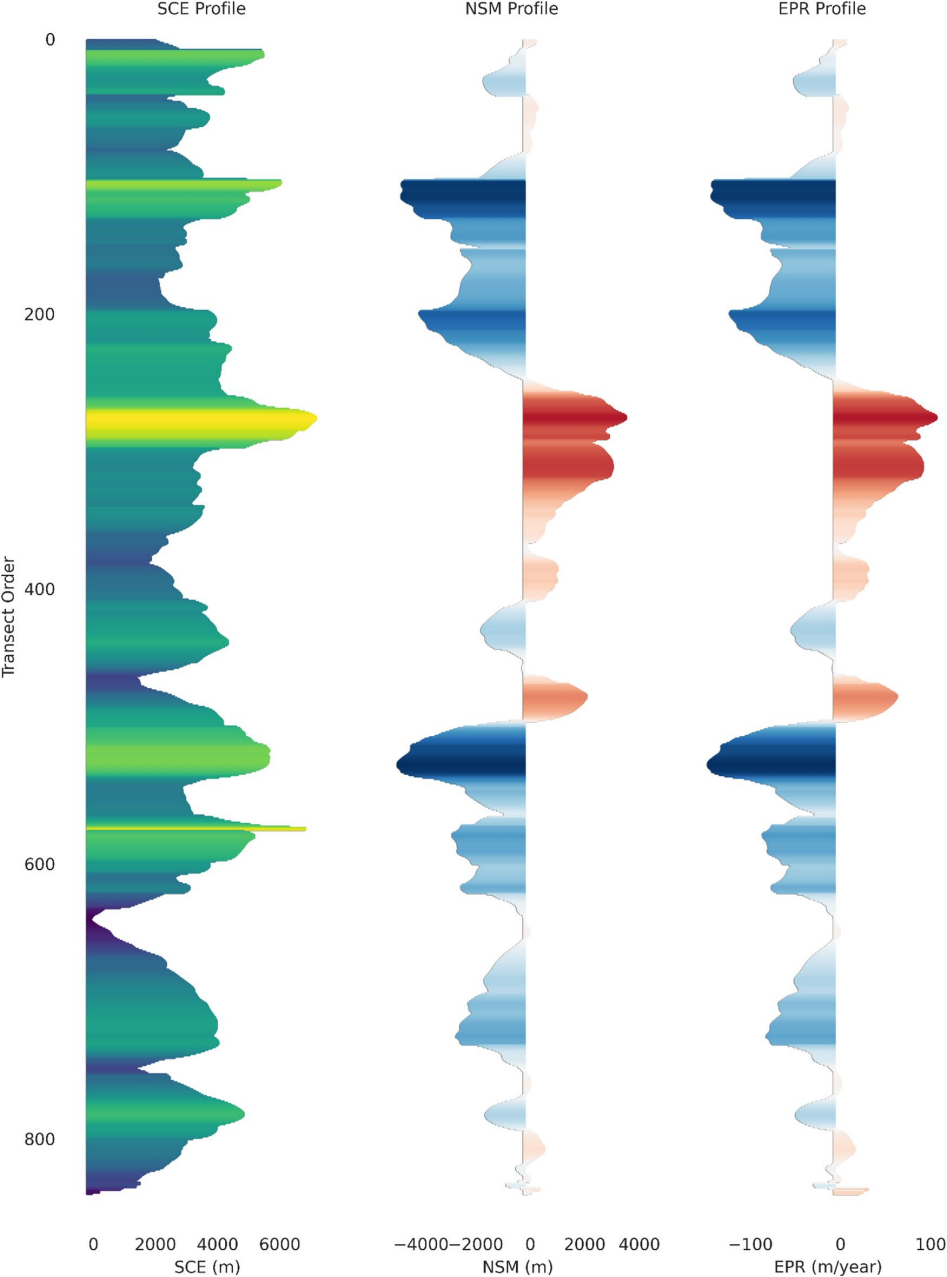
Transect-wise variation in right-bank shoreline dynamics along the Upper Ghaghara River (1991–2024). Metrics (SCE, NSM, EPR) were generated using DSAS v6.0 (https://code.usgs.gov/cch/dsas). Warmer colors (yellow–red) in SCE and NSM and EPR indicate higher magnitudes of bankline displacement and erosion, while cooler tones (purple/blue) represent lower change or accretion.

Figure 7 provides a transect-level analysis of these dynamics. The SCE profile reveals multiple peaks, especially between transect IDs 250 to 400, which correspond to zones of intense lateral bank migration seen in the spatial maps. The NSM bar chart indicates that the majority of transects (~ 63%) exhibit net erosion, with several transects showing retreat exceeding 2,000 m. The EPR profile similarly highlights concentrated zones of rapid erosion, with rates between − 100 and − 50 m/yr prevalent within these transects, while positive accretion rates up to + 100 m/yr occur in isolated segments. Together, these analyses demonstrate that the right bank of the Upper Ghaghara Riveris characterized by significant spatial and temporal variability, with mid-reach sections experiencing the most rapid and extensive erosion. This detailed characterization provides critical insight for river management, emphasizing the need for targeted intervention in the most vulnerable reaches.

### Soil erosion risk modeling and Spatial distribution of RUSLE factors

**Fig. 8:**
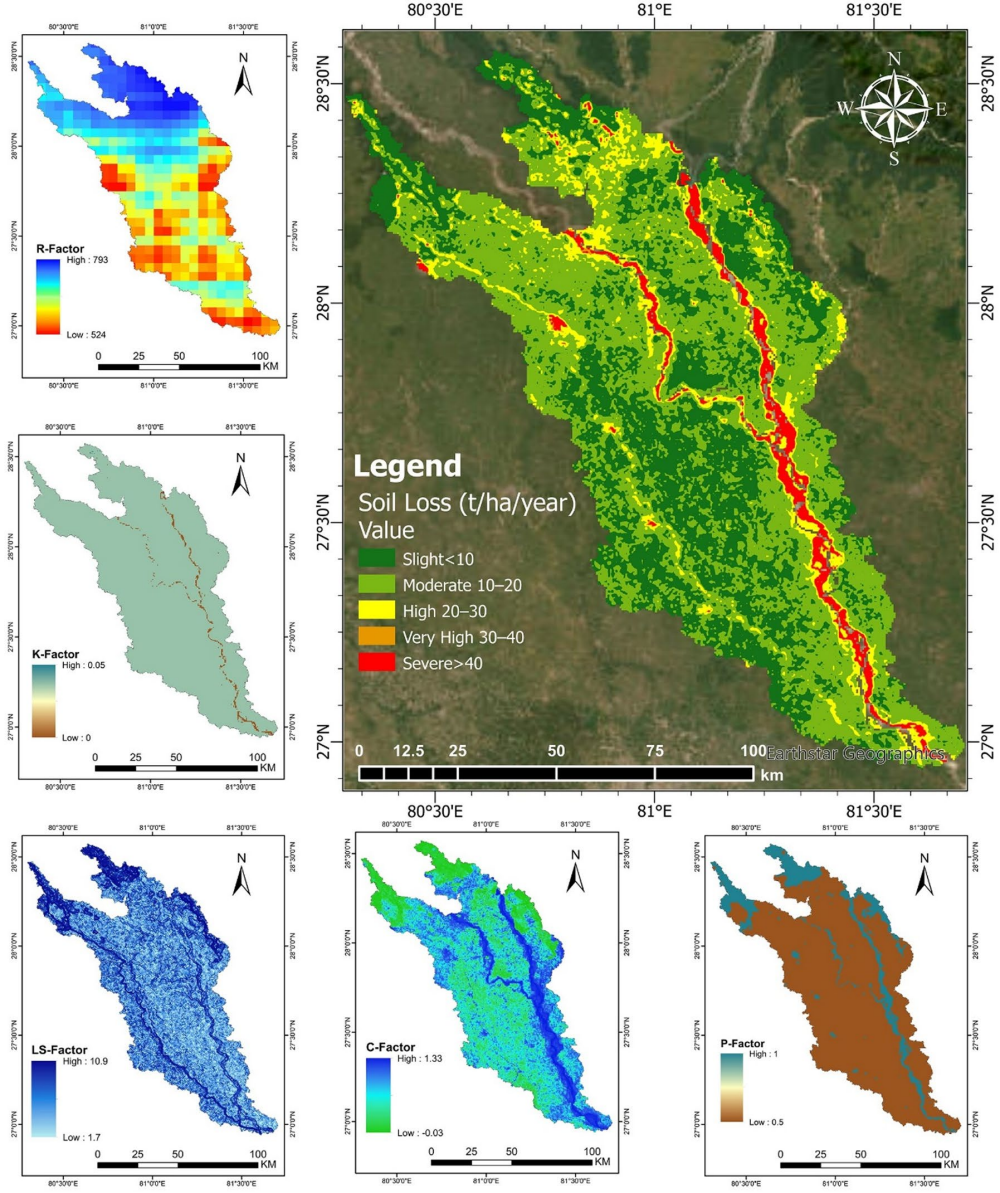
Spatial distribution of soil erosion risk and contributing factors in the Upper Ghaghara River Basin. Soil erosion modeling was performed in Google Earth Engine (https://earthengine.google.com/), and resulting maps were exported to ArcGIS Pro 3.2 (licensed version; https://www.esri.com/en-us/arcgis/products/arcgis-pro) for final map layout and symbology preparation.

The spatial assessment of soil erosion risk within the Upper Ghaghara River Basin was conducted using the RUSLE, integrating multiple erosion factors derived from remote sensing and field data (Fig. 8). The Universal Soil Loss Equation (USLE), first developed by Wischmeier and Smith (1978), was later refined into the RUSLE. This model is extensively used in different regions to estimate the loss of surface soil caused by rainfall, considering additional parameters such as soil characteristics and slope length^44^.

The soil loss map reveals a heterogeneous distribution of erosion risk across the catchment, with values ranging from slight (< 10 t/ha/yr) to severe (> 40 t/ha/yr). The highest erosion rates are concentrated along the active river corridors and steep slope areas, highlighted in red and orange, indicating severe to very high risk zones. These hotspots spatially coincide with regions of pronounced channel migration identified in the shoreline analysis.

The rainfall erosivity factor (R-factor) ranged significantly, from approximately 350 to 950 MJ mm ha^−^¹ h^−^¹ yr^−^¹. The highest erosivity values (> 800 MJ mm ha^−^¹ h^−^¹ yr^−^¹) were recorded primarily in southeastern regions, correlating strongly with areas experiencing higher rainfall intensities and frequent storm events. The northern and western regions exhibited lower R values (350–500 MJ mm ha^−^¹ h^−^¹ yr^−^¹), reflecting relatively less intense rainfall patterns. It shows higher values in the southeastern and central portions of the basin, reflecting spatial variability in precipitation intensity, which enhances erosive potential in those areas.

Soil erodibility (K-factor) varies according to soil texture and composition, with higher values concentrated in localized zones, indicating soils more susceptible to detachment and transport. Accordingly it varied notably across the basin, ranging from 0.15 to 0.45 t ha h ha^−^¹ MJ^−^¹ mm^−^¹. Soils characterized by higher sand and silt content, predominantly in the central and eastern regions, exhibited higher erodibility values (0.30–0.45). Areas with clay-rich soils demonstrated substantially lower erodibility values (0.15–0.25), primarily in the northern and western portions of the basin.

The slope length and steepness factor (LS-factor) highlights the influence of topography, where steeper and longer slopes in the basin’s hilly sections increase erosion susceptibility. It ranged broadly from 0.5 to 18. High LS values (> 10) were concentrated in hilly terrains, particularly in the central and southern parts, reflecting steep slopes and greater susceptibility to erosion. Lower LS values (< 3) were found predominantly on flat or gently sloping terrains, largely distributed in the basin’s northern and western areas.

The cover-management factor (C-factor) reflects land cover influence, showing reduced erosion potential in densely vegetated or well-managed areas, while bare or sparsely vegetated zones exhibit higher C-factor values. It ranged from 0.01 to 0.45. Higher values (> 0.3) were recorded in regions under intensive agricultural use with limited vegetation cover, significantly enhancing erosion susceptibility. Conversely, areas maintaining dense vegetation or implementing sustainable agricultural practices showed low C values (0.01–0.1), mainly located in protected or managed forested regions and sustainably cultivated lands throughout the basin.

Lastly, the support practice factor (P-factor) illustrates the effectiveness of field management practices, ranging from 0.1 to 1.0. Lower values (0.1–0.3) were observed in areas implementing effective erosion control measures, including terracing and contour farming. Regions lacking adequate conservation measures exhibited significantly higher values (0.8-1.0), identifying these as priority zones for targeted soil conservation interventions.

The combined assessment of all above factors resulted in estimated annual soil loss ranging from less than 5 t/ha/year in areas with low erosion susceptibility to greater than 40 t/ha/year in critical erosion zones. Approximately 60% of the basin exhibited soil loss below 10 t/ha/year, signifying relatively stable and sustainable conditions. However, around 25% of the basin faced moderate erosion (10–20 t/ha/year), and 10% experienced high erosion rates (20–40 t/ha/year). Critical zones with very high soil loss (> 40 t/ha/year) accounted for about 5% of the basin, highlighting urgent needs for implementing rigorous soil conservation and restoration measures to mitigate severe environmental impacts.

Together, these integrated maps demonstrate that the most severe erosion risks coincide with steep slopes, high rainfall intensity, and low vegetation cover, especially near active riverbanks where geomorphic processes drive landscape instability. This comprehensive RUSLE-based modeling highlights priority areas for soil conservation and riverbank stabilization to mitigate sediment yield and preserve watershed integrity.

### Flood susceptibility assessment of the upper Ghaghara river basin

**Fig. 9:**
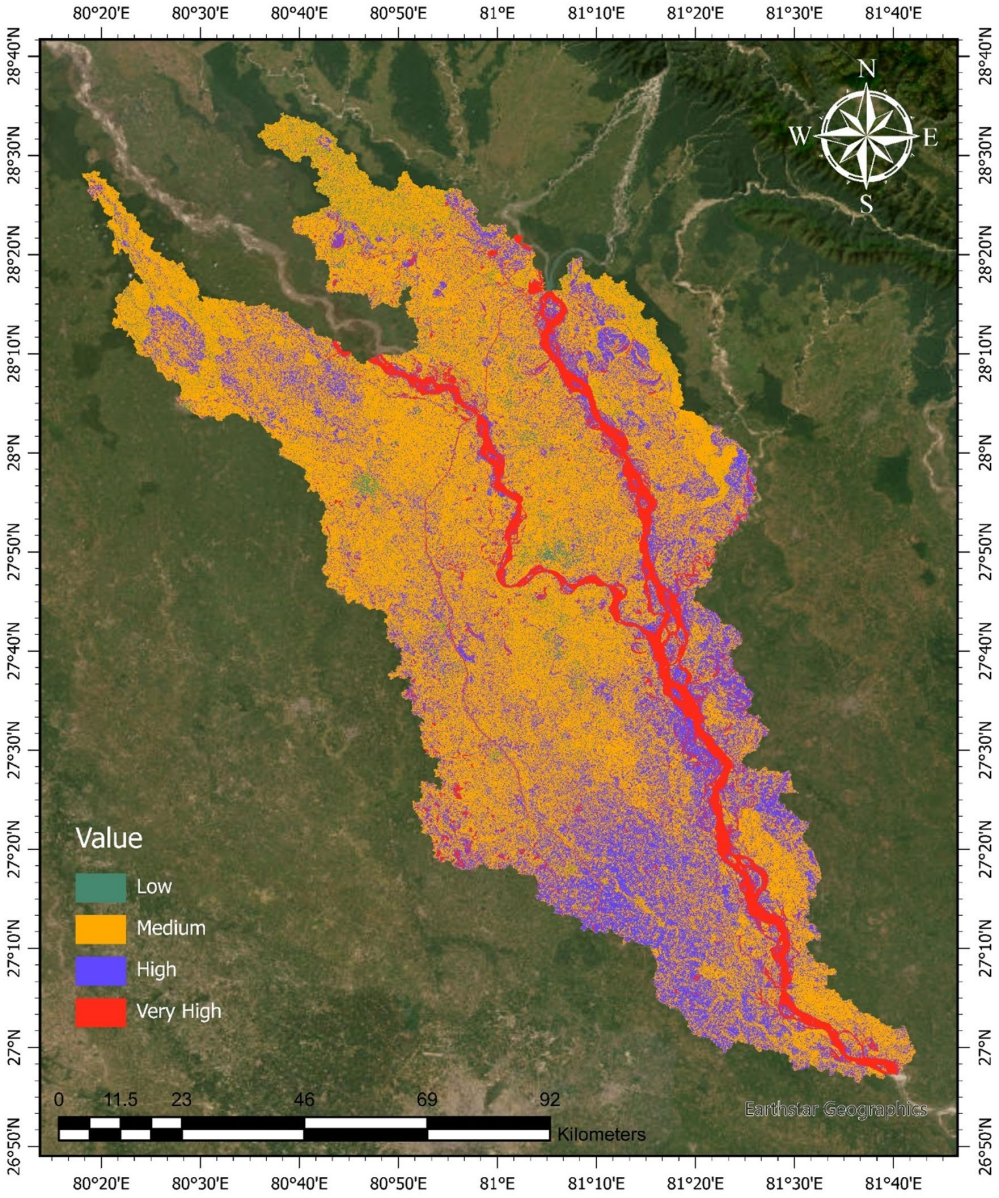
Flood susceptibility map of the Upper Ghaghara River Basin, categorized into four flood-risk zones: low, medium, high, and very high. Susceptibility analysis was conducted using an AHP-based method implemented in Google Earth Engine (https://earthengine.google.com), and map layout and symbology were prepared in ArcGIS Pro 3.2 (https://www.esri.com/en-us/arcgis/products/arcgis-pro/overview).

Figure 9 presents the flood susceptibility map of the Upper Ghaghara River Basin, delineating the spatial heterogeneity of flood risk based on a composite analysis of hydrological, topographical, and geomorphological parameters. The basin is classified into four susceptibility categories: low, medium, high, and very high, reflecting increasing likelihood and potential severity of flooding.

The medium susceptibility zone constitutes the predominant portion of the basin, encompassing extensive floodplain areas and low-relief terrains that experience recurrent, moderate inundation during seasonal high-flow periods. These zones typically exhibit gentle slopes and proximity to minor tributaries, making them susceptible to periodic waterlogging and overbank flooding. High susceptibility areas, shown in blue, are spatially associated with concave topographies, depressions, and floodplain margins adjacent to the main river channel, where hydrodynamic conditions favor frequent and sustained inundation. These zones represent critical interfaces between fluvial processes and terrestrial landscapes, characterized by elevated flood risk.

The very high susceptibility zones, highlighted in red, are primarily concentrated along the main river channels and their active floodplains, corresponding to geomorphologically dynamic segments prone to extensive flooding during peak discharge events. The AHP weighting procedure was internally consistent (CR = 0.06) and robust to sensitivity tests. Perturbing weights by ± 10% altered the extent of high and very high susceptibility zones by less than 5%, confirming that the derived flood susceptibility map is reliable and stable. These areas are of particular concern due to the compounded effects of channel migration, sediment deposition, and overflow, which can severely impact infrastructure and human settlements. Conversely, low susceptibility zones, marked in green, predominantly occur in upland regions with steeper slopes and higher elevations, where rapid surface runoff reduces flood retention potential, thereby lowering flood risk.

This flood susceptibility mapping integrates key physical drivers influencing flood occurrence, including terrain slope, drainage density, and hydrologic connectivity, providing a spatially explicit framework essential for risk-based land use planning and flood hazard mitigation. The delineation of flood-prone zones serves as a critical tool for guiding sustainable development, emergency preparedness, and targeted engineering interventions aimed at reducing flood vulnerability in the Upper Ghaghara basin.

### Flood mapping: pre- and post-flood analysis (2022)

Flood mapping was performed to assess the spatial extent and impact of flooding in the Upper Ghaghara River Basin during 2022^45^,. Two distinct time intervals were selected for analysis: a pre-flood period from March 1 to April 1, 2022, representing typical low-flow conditions, and a post-flood period from October 5 to October 13, 2022, corresponding to the peak high-flow phase. This temporal selection facilitates a clear comparison of flood-induced inundation dynamics.

**Fig. 10:**
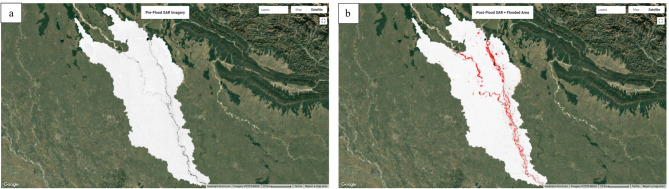
Flood extent mapping of the Upper Ghaghara River Basin during the 2022 monsoon season. (**a**) Pre-flood Sentinel-1 SAR imagery illustrates baseline water coverage. (**b**) Post-flood SAR imagery highlights newly inundated areas, indicating extensive flooding. Imagery and inundation analysis were processed using Google Earth Engine (https://earthengine.google.com), and final map layout was prepared in ArcGIS Pro 3.2 (https://www.esri.com/en-us/arcgis/products/arcgis-pro/overview). SAR datasets were sourced from the Copernicus Sentinel-1 archive (https://scihub.copernicus.eu).

The pre-flood map (Fig. 10a) depicts the baseline land surface conditions dominated by terrestrial land cover with minimal surface water. During this dry season, the landscape remained largely stable, with negligible inundation detected.

In contrast, the post-flood map (Fig. 10b) reveals extensive surface water coverage, indicating widespread flooding following the post-monsoon period. Floodwaters inundated large floodplain areas adjacent to the main river channels, visible as extensive waterlogged zones. The flooding closely follows the river network, with significant water retention in low-lying and flat terrains, consistent with regional hydrological patterns.

The comparative analysis of pre and post-flood imagery highlights a substantial increase in surface water caused by the flood event (Table 5). This information is vital for flood risk assessment, emergency response planning, and long-term watershed management. The detailed delineation of flooded zones provides key insights into the hydrodynamics of the Upper Ghaghara basin, emphasizing the need for targeted flood mitigation strategies in vulnerable areas.

**Table 5:** Flooded area summary (2022).

Flood Condition	Water Area (km²)
Pre-flood (Fig. 10a)	373.65
Post-flood (Fig. 10b)	743.09
Newly Flooded Area	483.53

While the flood extent comparison corroborates the modeled susceptibility zones, some mismatches at floodplain margins reflect limitations of SAR-based water detection and temporal offsets with peak flood timing. This underscores the need for integrating hydrodynamic simulations or field validation to refine future flood predictions.

## Discussion

The multi-decadal shoreline dynamics along the Upper Ghaghara River (1991–2024) show pronounced variability in both space and time. The right bank exhibits substantially higher erosion rates (EPR, NSM, SCE) compared to the left bank. This asymmetry has direct consequences for floodplain stability, the loss of agricultural land, and risk to riparian settlements. These findings affirm that localized management attention is needed in erosion-dominant reaches.

When soil erosion modeled via RUSLE is overlaid on bank migration data, we identify zones of dual hazard: areas where both upland soil loss and rapid bank retreat coincide. This duality intensifies downstream sediment flux, alters channel hydraulics, and accelerates morphological adjustments. Similar interactions have recently been documented in the Weyb River, Ethiopia, where land use practices and poor vegetation cover amplify vulnerability in erosion hotspots^22^.

In the context of early warning and mitigation, recent work by Shampa et al. (2025) presents an early warning system (EWS-RE) for riverbank erosion in the Brahmaputra–Jamuna braided river. That system integrates numerical hydro-morphological modeling with historical hydraulic data and bathymetry to predict erosion-prone zones with ~ 88% spatial accuracy^26^. This suggests that, beyond mapping, predictive tools could enhance the practical relevance of our results in the Upper Ghaghara, particularly in reaches showing frequent bank shifts.

Our results must be interpreted with regard to hydro-climatic cycles and climate change. Intensifying monsoon rainfall, increasing storm intensities, and potential glacial melt contributions are likely to exacerbate bank erosion and floodplain instability. While we used a simplified rainfall erosivity proxy (*R* = 0.5 × P), sensitivity tests in our study indicate that soil loss estimates vary by less than ~ 5% under moderate adjustment of weighting. We acknowledge that absolute soil loss magnitudes may be misestimated in certain settings without intensity data; thus, our findings are most reliable in terms of relative risk and spatial patterns rather than precise absolute values. In line with evidence from Southeast Asia that this simple formula can differ from intensity-based calculations by about 15–25%^38^, the totals changed, but the pattern of higher- and lower-risk areas stayed much the same. In practice, the maps are useful for comparing places (which areas face more erosion risk), while soil loss values, i.e. tons per hectare, should be treated as approximate and refined where intensity data are available.

Flood susceptibility mapping using AHP was validated with a low consistency ratio (CR = 0.06), and sensitivity analysis showed that variations in factor weights (± 10%) led to minimal changes in high and very high-risk zones. This robustness is supported by recent flood risk studies employing similar AHP approaches in Ethiopian and Bangladesh basins, which also emphasize the importance of consistency checks.

The consequences of erosion and flooding for livelihoods, agriculture, and infrastructure are increasingly evident. In the Jaldhaka River region, Alam et al. (2025) quantify substantial agricultural land loss from bank migration, reinforcing the urgency of protecting farmland in erosion-vulnerable regions. These socio-economic dimensions amplify the urgency of combining geomorphic risk mapping with land use planning, community engagement, and adaptive measures^42^.

During the September 2024 field visit, we collected qualitative, geo-referenced observations that corroborate active bank retreat and flood impacts. This study did not conduct systematic ground-truth field validation (e.g., RTK-GNSS bankline surveys, bathymetry, or plot-scale erosion measurements). This limitation may bias absolute rates (e.g., m yr^−^¹; t ha^−^¹ yr^−^¹), and therefore our results are most reliable for relative spatial patterns and hotspot identification rather than precise magnitudes. Future work should consider implementing RTK-GNSS/UAV surveys, ADCP cross-sections, plot-scale erosion monitoring, and event-based SAR/field verification to quantitatively validate and refine the DSAS–RUSLE–AHP outputs.

Taken together, this study shows that riverbank erosion, soil erosion risk, and flood susceptibility are interlinked. Though our methods rely on proxies in some components (e.g. rainfall erosivity), the spatial consistency of results, robustness tests, and similarity with findings in recent studies increase confidence in the main insights. The findings suggest that for the Upper Ghaghara Basin, practical efforts might include early warning systems, reinforcing riparian vegetation, buffer zoning, and applying predictive modeling to identify future hotspots. Such measures should be explored in collaboration with local stakeholders to ensure socio-ecological fit.

Beyond theory, river basins with comparable morpho dynamics have implemented field-tested measures that are transferable to the present study area i.e. Upper Ghaghara Basin.

(i) Setback/“Room-for-the-River”: move levees back, reconnect side channels, and lower parts of the floodplain to spread floods and allow safe lateral movement—this aligns with our high flood-susceptibility, migration-prone reaches. Dutch practice includes dike relocation, floodplain lowering, side-channel creation/reconnection, groyne lowering/realignment, removal of flow obstacles, high-water channels, and controlled depoldering (e.g., Noordwaard)^46^.
(ii) Targeted works at hotspots: short permeable groynes/spurs, toe protection, or limited revetments at top-quartile EPR/SCE clusters near critical assets for quick risk reduction, coordinated with spatial planning and local adaptation practice (as mainstreamed in Norwegian municipalities after extreme events)^47^.
(iii) Bioengineering and riparian vegetation: live staking, brush layering, and coir/log elements on moderate-energy bends and recent accretion zones to strengthen banks and reduce maintenance—evidence from peri-urban African catchments shows vegetation improves bank stability^48^.
(iv) Sediment-use controls: regulate in-channel sand mining and manage bars where bar growth coincides with EPR spikes to reduce thalweg swings and support other measures—demonstrated in the Mekong Delta^49^.

Across all items, monitoring and early warning (simple stage/velocity thresholds, periodic RTK/DSAS updates at known hotspots, community protocols) enable adaptive, low-cost roll-out^9,47^.

## Conclusion

This study presents a comprehensive analysis combining multi-decadal shoreline change (DSAS metrics including EPR, NSM, SCE), catchment-scale soil erosion risk (RUSLE), and flood susceptibility (AHP) in the Upper Ghaghara River Basin. The findings indicate that (i) the right bank has substantially higher erosion rates compared to the left bank; (ii) areas of rapid bank migration spatially overlap with zones of high modeled soil loss; and (iii) flood susceptibility classifications correspond generally with observed flood extents during the 2022 event.

However, when applying these findings and recommendations in other settings, consider (i) data (aligned image dates/water level, bankline accuracy, RUSLE factor checks, clean DEMs); (ii) resources (materials, native plants, design/O&M capacity, monitoring funds); (iii) policy/governance (permits, right-of-way, coordination); and (iv) geomorphic setting (sediment regime, planform, bank materials). Phased, low-risk pilot interventions at high-priority hotspots, are recommended with site checks and routine RTK/DSAS updates before scaling. Based on these data, several suggested pathways emerge that may help reduce geomorphic and flood hazards where they overlap, without asserting them as definitive policy mandates. These include:

- Implementing hybrid and bioengineering riverbank stabilization methods (e.g., live stakes, vegetative mats, brush layering, coir rolls) in highly erosive reaches. Studies elsewhere have shown that such methods can reduce bank erosion significantly while maintaining ecological functions^50^.
- Enhancing participatory watershed and floodplain land-use planning, including stakeholder involvement in mapping, buffer zone delineation, and restricting infrastructure or agricultural expansion near migrating banklines. Research from “Participatory approach to flood disaster management” shows that involving local communities improves flood risk outcomes and awareness^51^.
- Integrating shoreline change and flood susceptibility data into disaster preparedness frameworks, such as early warning systems, evacuation planning, and critical infrastructure siting. These approaches have been effective in regions where flood inundation and erosion risks are co-located.
- Improving upstream soil conservation practices (e.g., contour farming, enhanced vegetative cover, support practices) to reduce sediment supply to active bank migration zones, which may in turn reduce downstream bank retreat and floodplain sedimentation.

The study further suggests that future research efforts focus on evaluating the cost-effectiveness, ecological and social feasibility of these suggested interventions under local conditions. Additionally, improving model accuracy via inclusion of rainfall intensity metrics, climate change projections, and field validation of shoreline and flood inundation data would strengthen the ability to predict and plan for evolving risks.

## Supplementary Information

Below is the link to the electronic supplementary material.

Supplementary Material 1

## Data Availability

The datasets generated and/or analyzed during the current study are available from the corresponding author on reasonable request.
