# Supplementary material for: Quantifying riverbank and soil erosion risks in the upper Ghaghara river basin and their implications for flood management
Source: Sci Rep. 2026 Jan 16;16:3325. doi: 10.1038/s41598-025-33264-4 (PMC12835532; doi:10.1038/s41598-025-33264-4)
Supplement: Supplementary file 1 — Supplementary Material 1 [file 41598_2025_33264_MOESM1_ESM.docx]

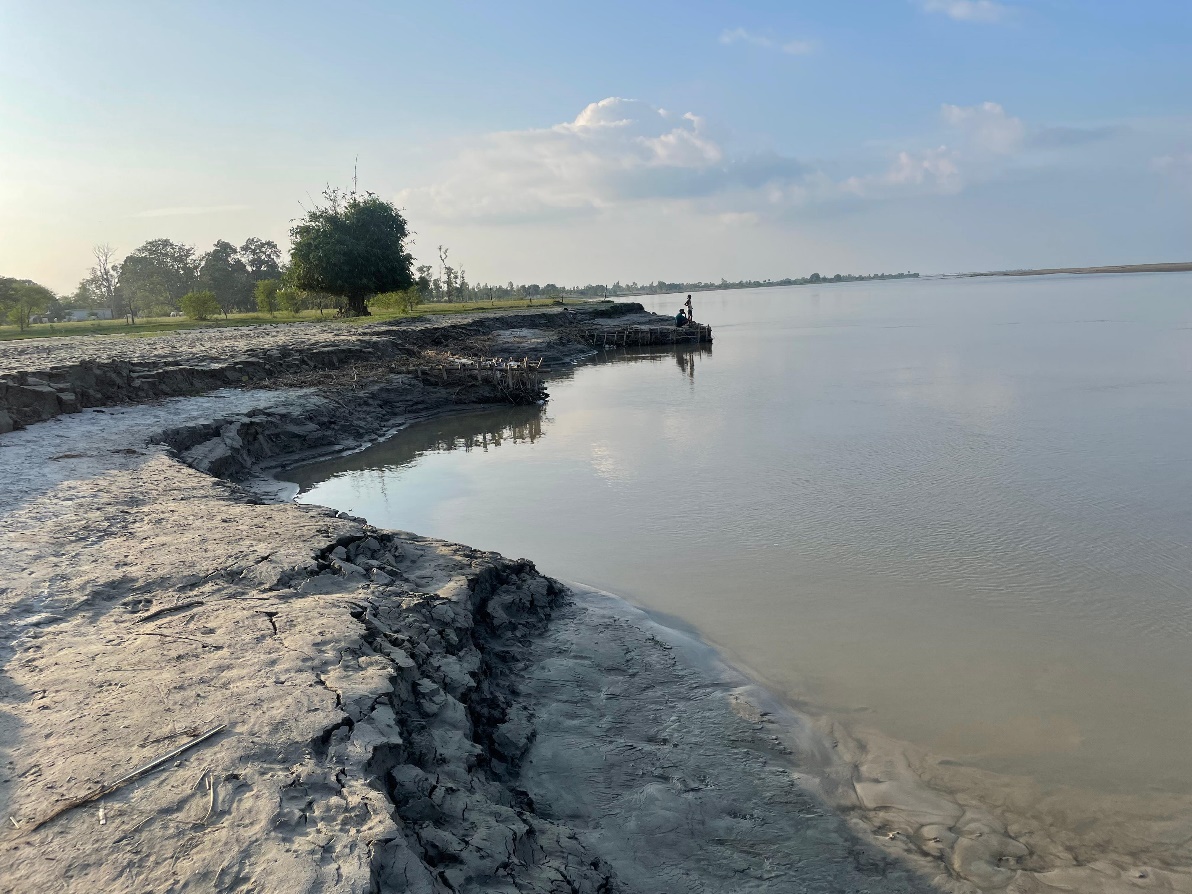


a


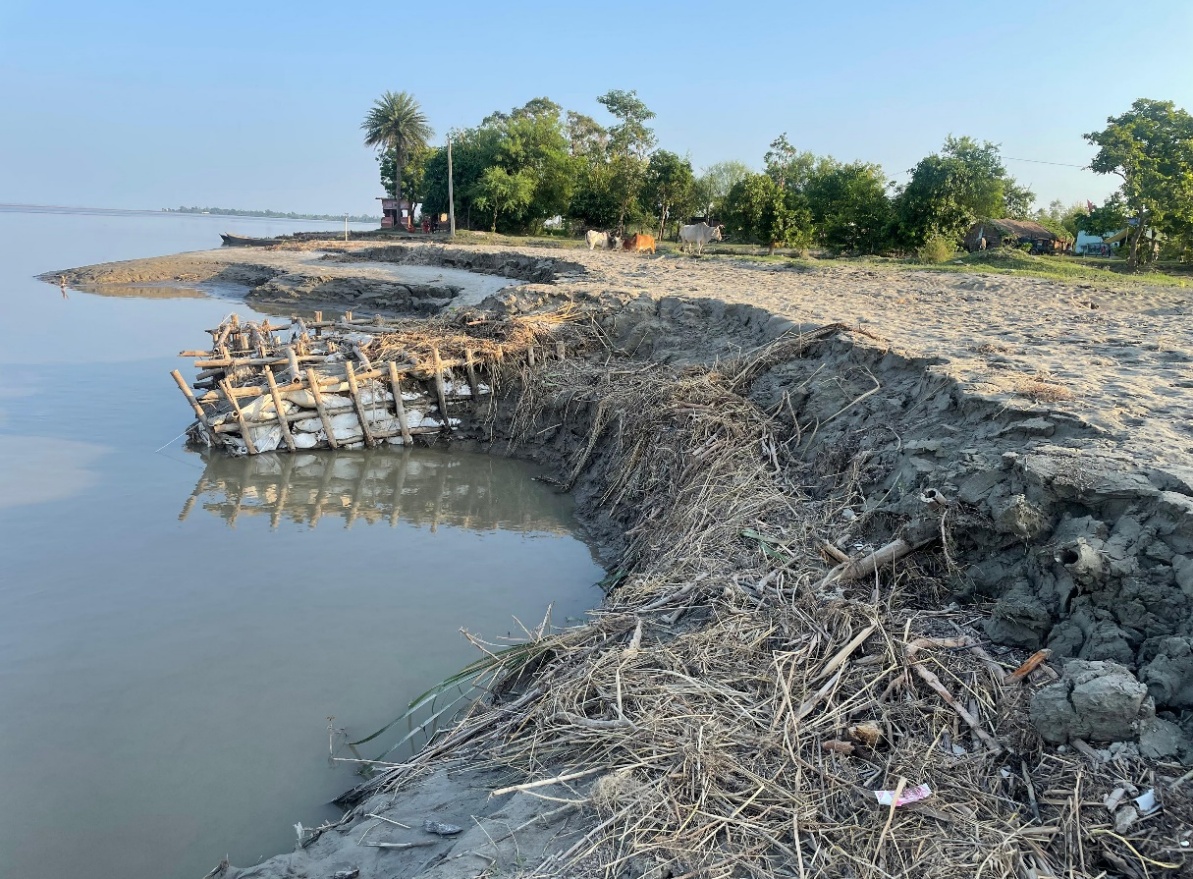


b

**Supplementary Figure S1.** Riverbank erosion observed near Village Gaudi, Bajaha, Block Reusa, showing significant bankline retreat and soil loss (photographs taken by the co-author, Santosh Kumar Pandey).

**(a)** Post‑monsoon (September 2024) view of bankline retreat and displaced soil.
**(b)** Detailed close‑up highlighting eroded bank edge, natural debris accumulation, and structural impacts.

| 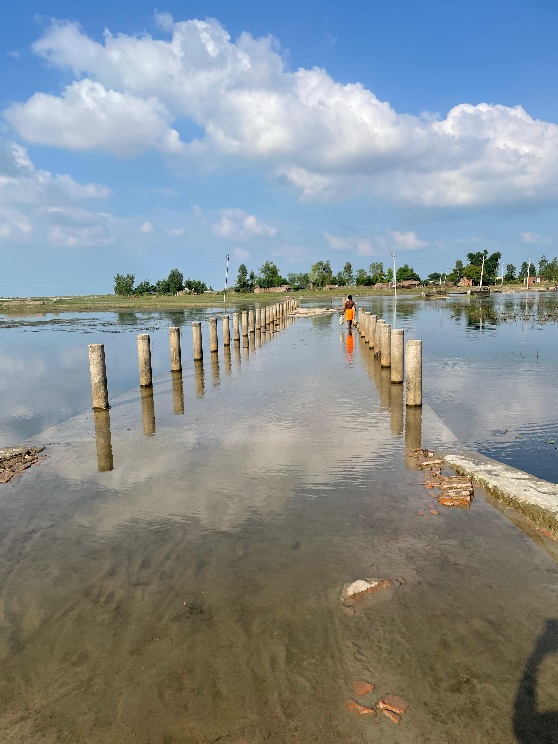  a | 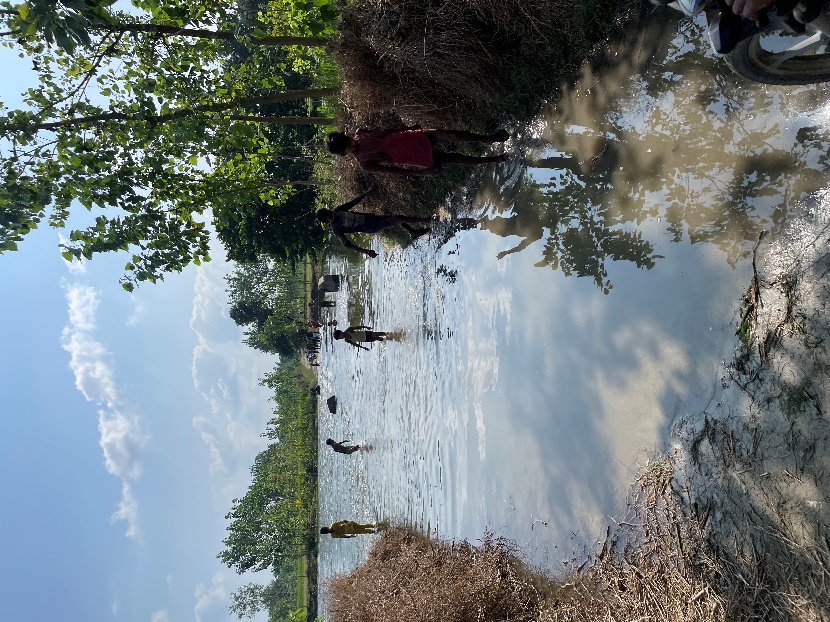  b |
| --- | --- |

**Supplementary Figure S2.** Flood impacts on infrastructure within the Upper Ghaghara basin (photographs taken by the co-author, Santosh Kumar Pandey).

**(a)** Flooded access road at Village Afsarya Husainpur (27°24′20.9″ N, 81°20′20.4″ E) showing complete cutoff during post‑monsoon flooding (September 2024).

**(b)** Overflowing stream on a bridge at Village Basantpur, Rampur Mathura (27°23′45.7″ N, 81°18′15.5″ E), illustrating floodwaters exceeding infrastructure capacity (September 2024).
